# Clade-Specific Recombination and Mutations Define the Emergence of Porcine Epidemic Diarrhea Virus S-INDEL Lineages

**DOI:** 10.3390/ani15152312

**Published:** 2025-08-07

**Authors:** Yang-Yang Li, Ke-Fan Chen, Chuan-Hao Fan, Hai-Xia Li, Hui-Qiang Zhen, Ye-Qing Zhu, Bin Wang, Yao-Wei Huang, Gai-Ru Li

**Affiliations:** 1 College of Animal Science, Anhui Science and Technology University, Chuzhou 233100, China; 2 State Key Laboratory for Animal Disease Control and Prevention, South China Agricultural University, Guangzhou 510642, China; 3 Guangdong Laboratory for Lingnan Modern Agriculture, College of Veterinary Medicine, South China Agricultural University, Guangzhou 510642, China

**Keywords:** PEDV, S-INDEL strains, mutation, recombination, evolution

## Abstract

Porcine epidemic diarrhea virus (PEDV) continues to circulate globally, causing substantial economic losses to the swine industry. Historically, PEDV strains are classified into the classical G1, epidemic G2, and S-INDEL genotypes. Among these genotypes, the highly virulent and prevalent G2 genotype has been extensively studied. However, recent clinical outbreaks in China necessitate a reevaluation of the epidemiological and evolutionary dynamics of circulating strains. This study analyzed 37 newly sequenced S genes and public sequences to characterize the genetic variations of S-INDEL strains. Our analysis revealed that S-INDEL strains are endemic throughout China, with a phylogenetic analysis identifying two distinct clades: clade 1, comprising early endemic strains, and clade 2, representing a recently dominant, geographically restricted lineage in China. While inter-genotypic recombination has been documented, our findings also demonstrate that intra-genotypic and intra-clade recombination events contributed significantly to the emergence of clade 2, distinguishing its evolutionary pattern from clade 1. A comparative analysis identified 22 clade-specific amino acid changes, 11 of which occurred in the D0 domain. Notably, mutations at positively selected sites—113 and 114 within the D0 domain, a domain associated with pathogenicity—were specific to clade 2. A phylodynamic analysis indicated Germany as the epicenter of S-INDEL dispersal, with China acting as a sink population characterized by localized transmission networks and frequent recombination events. These results demonstrate that contemporary S-INDEL strains, specifically clade 2, exhibit unique recombination patterns and mutations potentially impacting virulence. Continuous surveillance is essential to assess the pathogenic potential of these evolving recombinant variants and the efficacy of vaccines against them.

## Simple Summary

Porcine epidemic diarrhea virus (PEDV) S-INDEL strains, previously considered low-virulence, are now causing significant clinical outbreaks in China. Our study reveals that recently dominant S-INDEL variants (clade 2) in China evolved through unprecedented intra-genotypic and intra-clade recombination-distinct from recombination patterns observed previously. Crucially, mutations in the S protein’s D0 domain (sites 113/114), a region linked to a virulence determinant-induces structural alterations potentially enhancing pathogenicity, challenging prior virulence paradigms. Our findings underscore that continuous genomic surveillance of PEDV S-INDEL strains is essential to mitigate the risk of novel variants undermining existing control strategies and exacerbating economic losses in the pork industry.

## 1. Introduction

Porcine epidemic diarrhea (PED) is a highly contagious acute enteric disease caused by porcine epidemic diarrhea virus (PEDV). The infection manifests with the rapid onset of clinical signs including severe diarrhea, vomiting, and dehydration [1]. While PEDV affects swine of all age groups with varying clinical manifestations, infection in neonatal piglets is particularly severe, with mortality rates approaching 100% [2]. PED continues to pose a significant threat to global pork production and remains inadequately controlled despite ongoing efforts.

PEDV belongs to the genus *Alphacoronavirus* and the family *Coronaviridae*. It is an enveloped, positive-sense, single-stranded RNA virus with a genome of approximately 28 kb. Its genome encodes sixteen nonstructural proteins (nsp1–nsp16) derived from ORF1a/b polyprotein processing, four structural proteins—namely, spike (S), envelope (E), membrane (M), and nucleocapsid (N)—and an accessory protein-ORF3 [3,4]. Among these viral components, the S glycoprotein mediates virion attachment through receptor binding and serving as the primary target for neutralizing antibodies [5]. Due to its hypervariability, the S gene is widely utilized for assessing the genetic diversity of PEDV, elucidating epidemiological patterns, and guiding rational vaccine design [6].

Since its emergence in the 1970s, PEDV has undergone substantial genetic diversification during global dissemination, resulting in multiple strains with varying virulence and infectivity [7]. Phylogenetic classification delineates three major groups: classical (G1), epidemic (G2), and S-INDEL strains. G1 strains (G1a/G1b) exhibit lower virulence and affect swine populations sporadically [8,9]. The currently circulating G2 strains are further subdivided into G2a, G2b, and G2c subtypes, which are characterized by high pathogenicity and worldwide distribution [10,11]. S-INDEL strains, phylogenetically related to G1b and G2b strains, are believed to have emerged through recombination events between classical and mutant strains [12]. First identified in the United States in June 2013, their simultaneous detection suggests multiple parental introductions into North America [13]. Subsequent surveillance has confirmed their endemicity in the United States and Europe, with limited prevalence in Asia [13,14,15,16,17]. However, recent outbreaks of S-INDEL strains on Chinese farms [18,19], as confirmed in the present study, highlight the need for comprehensive epidemiological investigations of these strains, particularly within China.

Coronaviruses exhibit high mutation rates and recombination tendencies, enabling them to overcome host species barriers and immune defenses, thereby facilitating adaptation to new hosts or environments [20,21]. Recombination, a key evolutionary force driven by template switching during viral RNA replication, contributes significantly to coronavirus diversity by generating novel haplotypes, creating new epistatic interactions, and eliminating deleterious mutations [22,23,24]. In PEDV specifically, frequent recombination events have occurred and been documented in the following distinct contexts: (1) Inter-genotypic recombination: the exchange of genetic material occurs between strains belonging to different genotypes, such as between classical G1 and pandemic G2 strains [10,25,26]. This can lead to the emergence of viruses with novel combinations of traits. (2) Wild and vaccine strain recombination: genetic exchange is also observed between circulating wild-type strains and attenuated vaccine strains [23,27], potentially contributing to the evolution of viruses with altered pathogenicity or antigenicity. Alongside recombination, the continuous emergence of variant PEDV strains is also driven by mutations (including substitutions, insertions, and deletions) within the S protein [7,8,9]. Insertion and deletion mutations in the S protein are associated with the viral tissue tropism and pathogenicity of PEDV [8,11], for example, the amino acid insertion of the HNAY strain in the RBD domain of the S1 unit increased the infection of pigs [22]. Additionally, specific S protein mutations in the neutralizing epitopes of the S protein (e.g., mutations in G2 compared to G1, G2b compared to G2a) have been reported to be associated with cross-protection in vaccinated animals [10,28].

The emergence of G2 variant strains has attracted significant research attention not only in epidemiological and phylodynamic analyses, but also in mechanistic studies of PEDV. Motivated by recent S-INDEL strain outbreaks affecting pig farms and their distinct characteristics, we conducted a comprehensive investigation of these S-INDEL strains. Our study aimed to elucidate their evolutionary changes, thereby providing valuable insights for developing effective strategies to control and prevent PEDV circulation.

## 2. Materials and Methods

### 2.1. Detection and Amplification of PEDV S-INDEL Subtype S Gene

Specific primers and a probe for RT-qPCR-based detection of PEDV S-INDEL strains were designed using the PrimerQuest™ Tool (Integrated DNA Technologies, Coralville, IA, USA) based on conserved regions within the S protein sequences of known PEDV S-INDEL strains (GenBank accession numbers: KJ399978, KM392232, KR011756, KR003452, LM645057, KF453513, which have been confirmed by a previous study [29]) retrieved from the National Center for Biotechnology Information (NCBI) GenBank database (https://www.ncbi.nlm.nih.gov/, accessed on 1 September 2021). The oligonucleotide sequences were as follows: Forward: ATGTGCAGGCACCTGCTGTCG; Reverse: CGCCACTAGCAGTTTCAAGGC; Probe: FAM-ACTGTGGCACAGGCC-TAMRA, synthesized by General Biotechnology Co., Ltd. (Chuzhou, Anhui, China). Rectal swabs were collected from pigs exhibiting acute diarrhea across four Chinese provinces (Anhui, Guangxi, Jiangsu, Shandong) between 2021 and 2023. Specimens were suspended in phosphate-buffered saline (PBS), homogenized with vertexing, and centrifuged at 3000× *g* for 5 min at 4 °C. Supernatants were transferred to RNase-free tubes for viral RNA extraction using the Virus DNA/RNA Extraction Kit 2.0 (Vazyme (Nanjing, China), Cat. No. RM401). Complementary DNA (cDNA) was synthesized from the extracted RNA using a reverse transcription kit (Vazyme, Cat. No. R323) according to the manufacturer’s protocols. The samples were screened for S-INDEL strains via the designed RT-qPCR assay. Positive specimens underwent full-length S gene amplification using PCR with 2 × Taq Master Mix (Vazyme, Cat. No. P112-01) and established primers [30,31]. The amplicons were sequenced using the Sanger sequencing method by General Biotechnology Co., Ltd. (Anhui, China), yielding 37 complete S gene sequences (GenBank accession numbers: PV843383-PV843419).

### 2.2. Viral Sequence Collection

A total of 556 S gene sequences were analyzed in this study, including 37 newly sequenced samples. The remaining 519 sequences (comprising all available G1 and S-INDEL sequences, along with 80 reference G2 sequences which were confirmed by previous studies [10,32]) were obtained from the NCBI GenBank database on 10 March 2025. Sequence alignment was performed with MAFFT v7.526 [33] and inspected manually. The collection dates and locations of the S-INDEL strains were retrieved from NCBI GenBank and related references. Detailed information (including accession number and genotypes) of the sequences used in this study (both newly sequenced and publicly sourced strains) is provided in Table S1.

### 2.3. Preliminary Phylogenetic Analysis of PEDV S Gene

Phylogenetic analysis was performed using nucleotide sequences with IQ-TREE v2.2.0 [34] under the maximum likelihood (ML) framework. The best-fitting substitution model was identified using ModelFinder [35] based on Bayesian information criterion (BIC) values (GTR+F+R5 for 556 S gene ML tree, TIM+F+R4 for 415 S gene ML tree). The ML tree was assessed using the bootstrap method with 1000 replicates. An initial phylogenetic analysis of all 556 S gene sequences was conducted to distinguish S-INDEL strains. Subsequently, an ML tree of 415 S-INDEL strains was constructed to better understand their evolution. The resulting phylogenetic trees were visualized and annotated using the Interactive Tree Of Life (iTOL) platform (https://itol.embl.de/, accessed on 8 May 2025).

### 2.4. Amino Acid Analysis

Amino acid insertions and deletions distinguishing S-INDEL from non-S-INDEL strains were analyzed following previously established methods [7]. Additionally, phylogenetic clade-specific amino acid variations within S-INDEL strains were identified using Jalview v2.11.4.1 [36] and subsequently visualized using Python 3.12.9 with pandas v2.2.3 [37], Logomaker v0.8.7 [38], Matplotlib v3.10.1 [39], and the Numpy v2.2.4 [40] library.

### 2.5. Recombination Analysis

Potential recombinant events within the S gene were systematically screened using RDP v5.67 [41] with the default parameters. Seven recombination detection algorithms (GeneConv, BootScan, Chimaera, SiScan, RDP, MaxChi, and 3Seq) were employed to characterize recombinant events, including the identification of putative parental strains and breakpoint localization. A consensus threshold of at least three concordant methods with statistical significance (*p* < 0.01, Bonferroni-corrected) was applied to confirm valid recombination events. The procedure was repeated until no additional recombination events were detected. Potential recombination breakpoints within S-INDEL strains were further analyzed using Genetic Algorithm for Recombination Detection (GARD) [42]. Phylogenetic discordance was assessed via constructing separate maximum likelihood trees (1–1245 nt and 1246–4161 nt ML trees) for breakpoint-flanking regions to validate recombination, which was performed using R 4.4.2 (R Core Team (Vienna, Austria), 2024) scripts [43]. In detail, the substitution models for the 1–1245 nt and 1246–4161 nt ML trees were TIM+F+R4 and TIM+F+I+I+R3, respectively, and the final ML trees were assessed using the bootstrap method with 1000 replicates.

### 2.6. Selective Pressure Analysis

Selection analysis of the S gene in the non-recombination S-INDEL strains (final dataset including 243 sequences) was performed using the Datamonkey webserver (https://www.datamonkey.org/, accessed on 8 April 2025). Four methods were employed to detect the selection at individual sites: Mixed Effects Model of Evolution (MEME) [44], Fixed Effects Likelihood (FEL) [45], Single-Likelihood Ancestor Counting (SLAC) [45], and Fast, Unconstrained Bayesian AppRoximation (FUBAR) [46]. Statistical thresholds were set as follows: *p* < 0.05 for MEME, FEL, and SLAC, and posterior probability (PP) > 0.9 for FUBAR. The sites selected positively through consensus were defined as those concurrently identified using at least three independent methods.

### 2.7. Molecular Modeling and Structural Analysis of S-INDEL S Protein

Consensus amino acid sequences for each S-INDEL clade were generated using Jalview [40] to investigate functional mutations in the S protein. The tertiary structures of the S protein for each clade were predicted using the SWISS-MODEL homology modeling server (https://swissmodel.expasy.org/, accessed on 13 April 2025) with the pdb file 7W6M.1 as the structural template. Structural comparisons and visualizations were performed using PyMOL software (The PyMOL Molecular Graphics System, Version 3.1 Schrödinger LLC, New York, NY, USA).

### 2.8. Evolutionary Dynamic Analysis of PEDV S-INDEL Strains

Following the removal of all recombinant sequences, a maximum likelihood (ML) phylogeny was constructed from the filtered dataset using default settings with Ultrafast Bootstrap approximation [47] with 1000 replicates. The best-fitting substitution model (TIM+F+I+I+R3) was identified with ModelFinder [35] based on the BIC values. The resulting topology was analyzed using TempEst v1.5.3 [48] to assess the temporal signal consistency. Evolutionary dynamics parameters were estimated using BEAST v1.10 [49] with a relaxed uncorrelated lognormal (UCLN) molecular clock model and a Bayesian skyline coalescent model [50] designated as the tree prior. Markov chain Monte Carlo (MCMC) analyses were run for 5 × 10^8^ generations, sampling parameters every 5000 generations to ensure computational convergence. The effective sample size (ESS) values for all parameters exceeded 200, as verified using Tracer v1.7 [51]. Following a 10% burn-in, two independent runs were combined using Logcombiner v1.10 [49]. Final maximum clade credibility (MCC) trees were generated with TreeAnnotator v1.10 [49] and visualized by Figtree v1.4.4 (http://tree.bio.ed.ac.uk/software/figtree/, accessed on 20 May 2025).

### 2.9. Discrete Phylogeographic Analysis of PEDV S-INDEL Strains

Discrete phylogeographic inference was performed using BEAST v1.10 [49] with sampling countries assigned as discrete state traits. Nineteen discrete locations (including Australia, Belgium, Canada, Colombia, China, Croatia, France, Germany, Italy, Japan, Mexico, Netherlands, Poland, Romania, South Korea, Slovenia, Spain, USA, Vietnam) were involved in this analysis. A symmetric substitution model with Bayesian Stochastic Search Variable Selection (BSSVS) was implemented to model dispersal dynamics and identify statistically supported transition rates between locations. Statistically significant migration routes (Bayes factor > 3) were evaluated using SpreaD3 v0.9.7 [52]. The number of state counts of the migration in and out of each discrete location (Markov jump counts and rewards) [53] were calculated to quantify spatial transitions across discrete locations. Two independent chains of 5 × 10^8^ generations (sampled every 50,000 generations) were run, following the first 10% burn-in. Computational efficiency was enhanced using the BEAGLE v3.1.0 high-performance library [54].

## 3. Results

### 3.1. Phylogenetic Analysis of PEDV S-INDEL Strains

**Figure 1:**
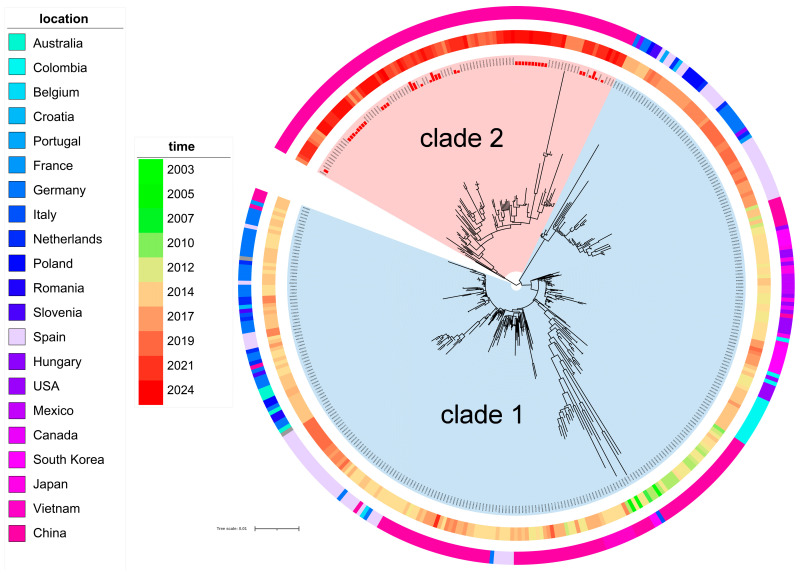
The maximum likelihood phylogeny of PEDV S-INDEL strains. The phylogenetic tree of S-INDEL strains with 2 clades was classified. The strains sequenced in this study are highlighted in red at the tips. Geographic isolation and the collection years are indicated by outer ring colorations.

To elucidate the evolutionary relationships of PEDV S-INDEL strains, we first constructed a maximum likelihood (ML) phylogeny to differentiate S-INDEL from non-S-INDEL strains. The analysis revealed that S-INDEL strains formed an independent clade (Figure S1). Compared to non-S-INDEL subtypes, the S-INDEL genotype exhibited a main deletion within amino acid residues 55–65 (SMN----SSSW) and an insertion within residues 171–172 (GK). Based on these molecular signatures and phylogenetic topology, we subsequently constructed an ML tree of S-INDEL strains, which resolved into two distinct clades, with the bootstrap value in each clade being 100 (clade 1 and clade 2; Figure 1). The 37 newly sequenced strains from this study exclusively clustered within clade 2. Furthermore, analysis of the sample locations and collection times revealed that clade 1 strains predominantly circulated in China, Germany, Spain, and the United States before 2019, with only one sequence detected since 2021, while all clade 2 strains were detected in China after 2019, indicating distinct epidemiological patterns between the two clades.

### 3.2. Recombination Analysis of PEDV S-INDEL Strains

**Figure 2:**
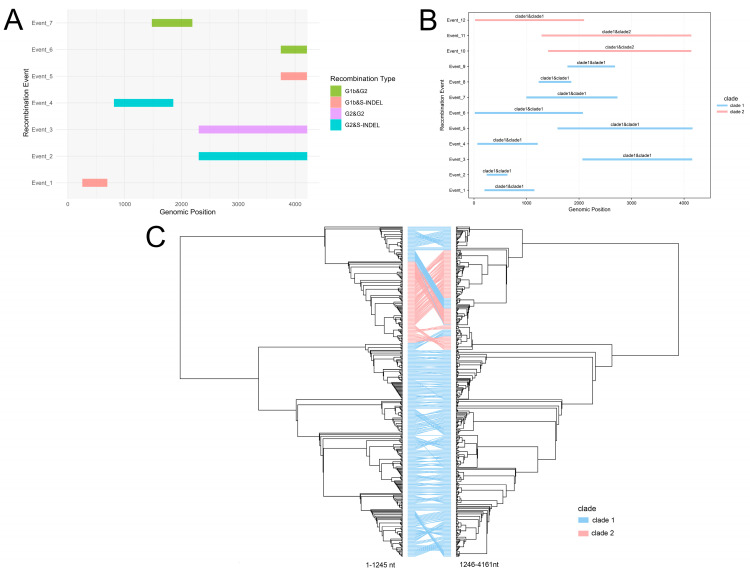
The recombination analysis of PEDV S-INDEL strains. (**A**) Inter-genotypic recombination events. Each event is colored according to donor/recipient pairs. (**B**) Intra-genotypic recombination between S-INDEL clades. Events are annotated to show inter-clade recombination patterns between clade 1 and clade 2. (**C**) The phylogenetic incongruence analysis. Maximum likelihood trees constructed from genomic segments (1–1245 nt, 1246–4161 nt) flank the predicted recombination breakpoints identified with GARD.

Given previous reports suggesting that S-INDEL strains represent G1/G2 recombinants, we performed comprehensive recombination analyses. First, the RDP5 analysis of 556 sequences identified different kinds of recombinant events among S-INDEL strains, including both inter-genotypic and intra-genotypic recombination across different genomic regions (Figure S2). To characterize parental genotypes, we analyzed putative parental sequences according to clade/genotype (Tables S2 and S3). Inter-genotypic recombination analysis based on the parental sequences belonging to each genotype revealed that four distinct patterns were involved: G1b&G2, G2&G2, G1b&S-INDEL, and G2&S-INDEL (Figure 2A, Table S2). Regarding intra-genotypic recombination within S-INDEL strains, analysis indicated that clade 1 recombinants primarily resulted from recombination between clade 1 strains, while clade 2 recombinants originated from both clade 1 and clade 1 recombination, and clade 1 and clade 2 recombination (Figure 2B). Following recombinant exclusion, we determined that clade 2 itself constitutes a recombinant lineage. Furthermore, GARD analysis detected one breakpoint among the S-INDEL strains, with alignment position 1245 representing a statistically significant position, then the co-phylogenetic analysis of the 1–1245 nt and 1246–4161 nt ML trees revealed frequent incongruence among the S-INDEL clade 1 strains, and no congruence in the topology of clade 2 (Figure 2C), which was consistent with the RDP5 analysis that showed frequent recombination among S-INDEL strains, especially clade 2.

### 3.3. Comparison of Amino Acid Mutations in S Protein Between Clade 1 and Clade 2 of S-INDEL Strains

**Figure 3:**
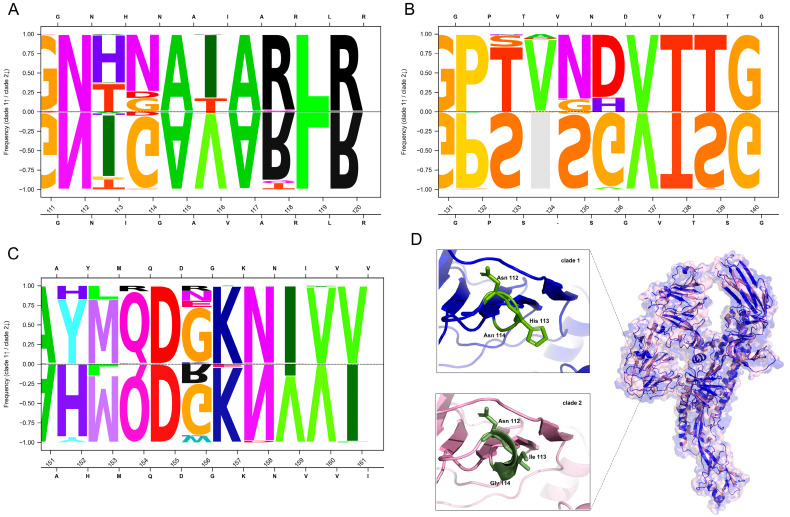
The comparative analysis of amino acid variations between S-INDEL clade 1 and clade 2. The seqLogo comparison of part of the D0 domain between clade 1 and clade 2. The differential residue frequencies at positions (**A**) 111–120, (**B**) 131–140, (**C**) 151–161. *X*-axis: the amino acid position and identity; *Y*-axis: the occurrence frequency (%) per clade. (**D**) The predicted structural models of the spike protein, comparing clade 1 (blue) and clade 2 (light pink). Magnified views highlight the functionally critical regions, residues 112–114, which belong to the D0 domain.

Comparative amino acid analysis revealed that most amino acid differences between clade 1 and clade 2 were primarily localized to the S1 unit (Figure S3). Specifically, in the D0 domain, ten amino acid substitutions and one deletion in residue 134 were observed compared with clade 1 (Figure 3A–C); in the NTD, eight substitutions were observed. Apart from these mutations, another three substitutions also occurred in the signal sequence (SS) domain. The detailed alterations in the S protein were as follows: 5 (N→T), 28 (S→L), 30 (I→T), 113–114 (HN→IG), 116 (I→V), 133–136 (TVND→S-SG), 139 (T→S), 152 (Y→H), 159 (I→V), 161 (V→I), 232 (S→I), 242–243 (DS→EP), 266 (L→V), 300 (M→I), 309 (A→V), 338 (L→F), 351 (D→N) (Figure S3). Structural modeling identified the HN→IG substitutions at residues 112–114, which induced helix formation in clade 2 strains (Figure 3D).

### 3.4. Analysis of Positive Selection Sites in S-INDEL Strains

**Figure 4:**
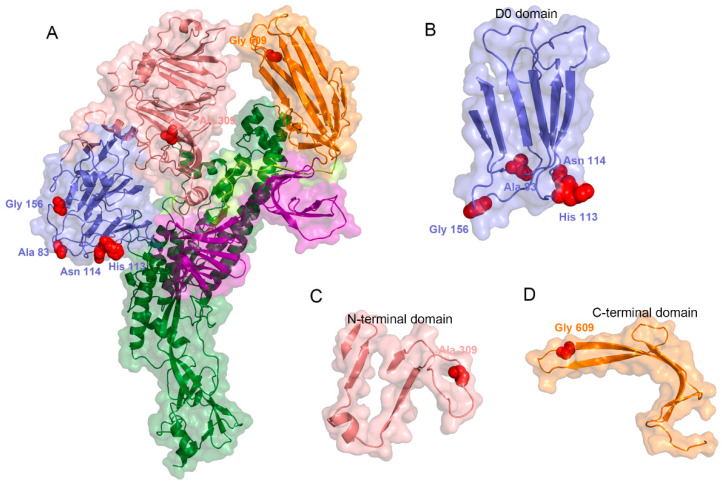
Structural mapping of the positively selected residues in PEDV spike protein variants. (**A**) The full-length spike protein model with positively selected residues indicated by red spheres. Different colors represent domain of spike protein (blue represent D0 domain, salmon represent N-terminal domain, limon represent SD1 domain, orange represent N-terminal domain, purple represent SD2 domain, and forest represent S2 unit ) (**B**–**D**) Magnified views of the selected hotspots in functional domains: (**B**) the D0 domain, residues 83, 113, 114, 156; (**C**) the N-terminal domain (NTD), residue 309; (**D**) the C-terminal domain (CTD), residue 609.

Analysis of non-recombinant S-INDEL strains identified eight codons under positive selection (positions 10, 83, 113, 114, 156, 309, 609, and 1376; Table S4) which were identified with at least three distinct methods. Integration of these findings with the S protein homotrimer structure enabled the mapping of six positively selected codons (Figure 4A), which localized to three distinct structural domains: codons 83, 113, 114, and 156, located on the surface of the D0 domain (Figure 4B); codon 309, located on the surface of the N-terminal domain (NTD) (Figure 4C); and codon 609, located on the surface of the C-terminal domain (CTD) (Figure 4D), which also belongs to the core neutralization epitope region (COE), a prominent target for subunit vaccines under development to prevent viral infection.

### 3.5. Evolutionary Dynamics Analysis of S-INDEL Strains

**Figure 5:**
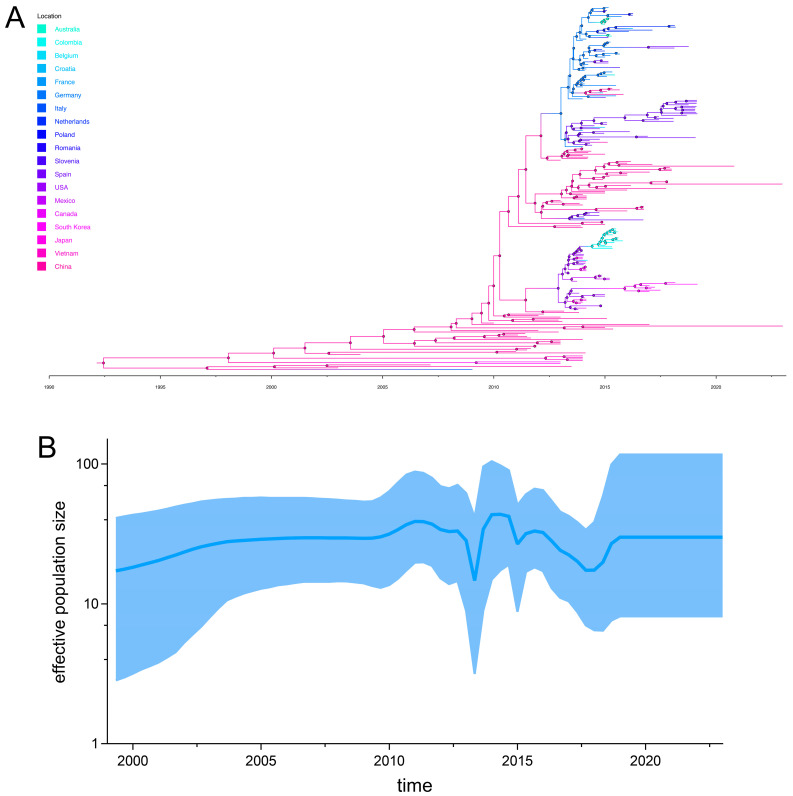
The maximum clade credibility (MCC) phylogeny of PEDV S-INDEL strains. (**A**) Branches are colored according to the country of collection. (**B**) The temporal changes in effective population size, with the mean value represented by the blue line and the 95% credible intervals (CI) indicated by the blue shaded area.

To mitigate the confounding effects of recombination on phylodynamic reconstruction, recombinant S-INDEL strains were excluded from this analysis. Temporal signal assessment of the non-recombinant S-INDEL strains revealed a strong clock-like signal (R^2^ = 0.48), supporting the suitability of these data for time-calibrated phylogenetic reconstruction. Maximum clade credibility (MCC) trees demonstrated that European and American strains formed distinct clades (Figure 5 and Figure S4). The mean evolutionary rate of S-INDEL strains was 1.73 × 10^−3^ substitutions/site/year (95% HPD: 1.52 × 10^−3^–1.93 × 10^−3^), with a tMRCA estimated at 1991.2 (95% HPD: 1980.5–1999.1). Bayesian skyline analysis showed that the effective population size of S-INDEL strains underwent gradual expansion starting in 1999.3, reaching peaks in 2011 and 2014, with intermittent fluctuations during this period (Figure 5B).

### 3.6. Spatial Dynamics of PEDV S-IINDEL Strains

**Figure 6:**
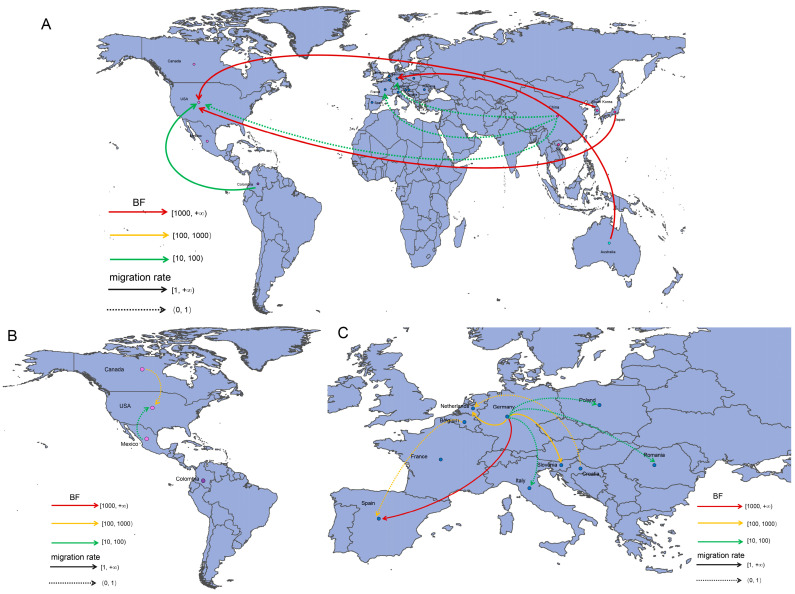
A discrete phylogeographic reconstruction of PEDV S-INDEL strain dissemination. Migration pathways are colored according to their Bayesian factor (BF) support strength: green: 10 ≤ BF < 100 (supported), orange: 100 ≤ BF < 1000 (strong), red: BF ≥ 1000 (very strong). Line styles indicate the migration rates: dashed: 0 < rate < 1, solid black: rate ≥ 1. (**A**) Intercontinental transmission routes. (**B**) Intra-American country diffusion. (**C**) Intra-European country diffusion.

**Figure 7:**
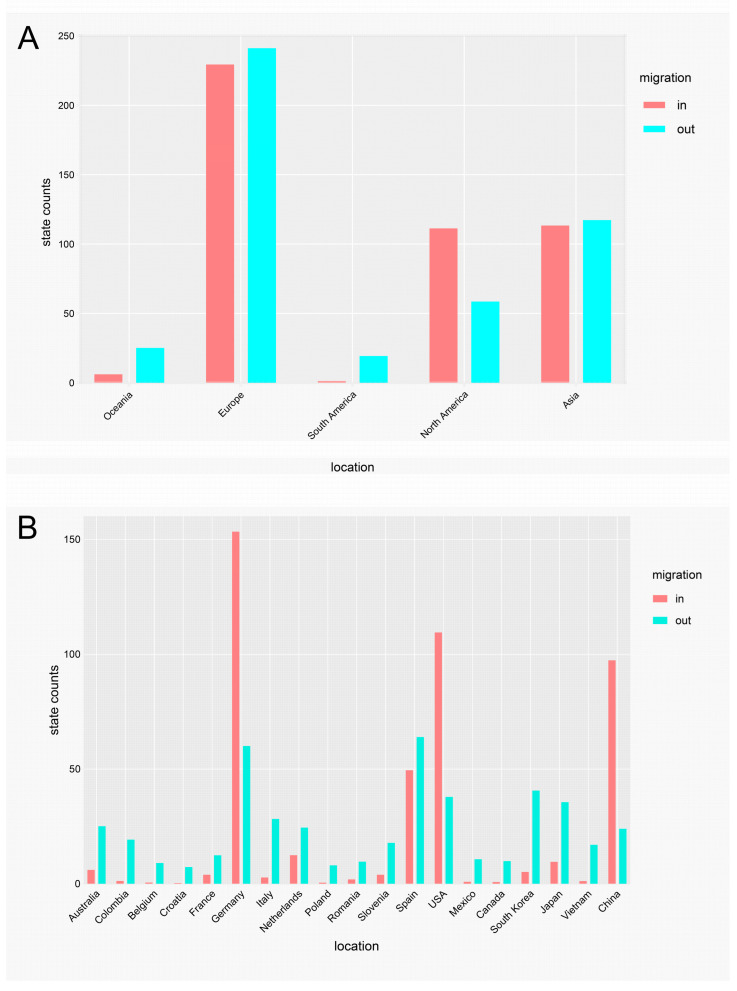
The Markov jump counts and returns for PEDV S-INDEL strains. Pink bars represent migration in counts; light green bars represent migration out counts. (**A**) The continental level. (**B**) The individual country level.

The discreate phylodynamic analysis across 19 countries identified seventeen well-supported migration pathways (BF > 10; Figure 6). Six pathways originated from various regions and terminated in the United States (US). Six pathways originated from Germany and terminated in other regions. Notably, four migration links exhibited exceptionally strong support (BF > 1000): from Australia to Germany, from Germany to Spain, from Japan to the US, and from South Korea to the US. Furthermore, a comparison of migration rates identified the pathway from Germany to Spain as having the highest rate (2.39). Markov jump counts and Bayes factor reward analyses further indicated that outward migration from Europe, particularly from Germany and Spain, was dominant; Asia, especially South Korea and Japan, also plays an important role (Figure 7). Inward migration to Europe, especially to Germany, was also prominent. Additionally, the US in North America and China in Asia were identified as significant recipient countries for viral migration. Collectively, these findings indicate that Germany served as the primary source population for the global dissemination of S-INDEL strains.

## 4. Discussion

PEDV continues to challenge the global pork industry, despite widespread vaccination programs and enhanced biosecurity measures. While studies have predominantly focused on non-S-INDEL strains, due to their higher virulence in pigs [55,56,57], recent outbreaks of clinical diarrhea in pigs caused by S-INDEL strains in China have underscored the need to investigate their epidemiological significance. Our study addresses a critical knowledge gap by systematically investigating the S-INDEL strains in China—variants distinct from previously reported S-INDEL strains. The following key findings warrant emphasis:

Firstly, the epidemiological shift of S-INDEL strains necessitates surveillance. Our 2021–2023 surveillance across four Chinese provinces confirmed the endemicity of PEDV S-INDEL strains in China, with recent outbreaks causing significant mortality [18,19]. Furthermore, the phylogenetic reconstruction of S-INDEL strains revealed two distinct clades with unique geographical distributions and temporal emergence patterns. Notably, clade 2, which predominantly comprises strains from recent Chinese strains, might have displaced early clade 1 strains predominant in the United States and Europe, mirroring observations from the United States that early epidemic clades failed to establish persistent lineages [13]. These findings underscore the importance of monitoring the prevalence of and changes in S-INDEL strains, despite previous reports suggesting lower pathogenicity and transmission efficiency compared to non-S-INDEL strains [58].

Secondly, mutations of residues 113 and 114 in the D0 domain might implicate virulence evolution. Coronavirus S proteins are reported to contain various functional amino acids, and changes in specific critical amino acids can significantly affect viral infectivity [59]. Wen and Su et al. have found that the genetic mutation of S1 is increasing over time [60,61,62]. Our comparison analysis revealed 22 clade-specific amino acid mutations that distinguish clade 2 from historical clade 1 S-INDEL strains. Furthermore, the mutations are predominantly localized to the D0 domain (*n* = 11) and NTD (*n* = 8). The D0 domain mediates sialic acid binding and serves as a virulence determinant in PEDV, and S62 mutation in the D0 domain has been reported to affect viral virulence [63]. Our study revealed that D0 domain mutations at S113 and S114 are identified as positive sites during their evolution. Therefore, whether the clade-specific mutations at positions 113 and 114 can alter the virulence of clade 2 strains requires further confirmation using reverse genetic techniques. Additionally, positive site 609 within the core neutralization epitope region contains a major neutralizing epitope and is a prominent target for subunit vaccines under development [64,65,66,67], suggesting potential implications for vaccine efficacy.

Thirdly, the recent clade 2 S-INDEL strains have formed a recombinant lineage. While S-INDEL strains were originally proposed as recombinants between classical strains and mutant strains [10], and the United States variants primarily resulted from inter-strain recombination [13], Wang et al. also found evidence of inter-strain recombination in the S-INDEL strains they sequenced [19]. Unlike what was previously reported, our analysis revealed both inter- and intra-strain recombination events contributing to S-INDEL evolution, particularly the recent clade 2 strains that formed a recombinant lineage. These may stem from the larger number of S-INDEL strains analyzed in this study, especially the inclusion of more recent Chinese sequences since 2021. Frequent recombination events among Chinese S-INDEL strains and increasing mutations during evolution might indicate that the emergent recombinant clade 2 in China is forming localized transmission networks. Therefore, recombinant clade 2 warrants further investigation.

Overall, our integrated analysis of historical and contemporary S-INDEL variants reveals that the emergent clade 2 represents a recombinant lineage with distinct genetic features. Compared to clade 1 (historically endemic strains), clade 2 exhibits critical mutations at sites 113 and 114, which have been found to be positive sites. Although S-INDEL strains have traditionally been considered low-pathogenicity variants, the pathogenic potential and vaccine susceptibility of these recombinant strains require urgent evaluation given their recent expansion in Chinese swine populations.

## Data Availability

The authors confirm that the data supporting the findings of this study are available within the article and its Supplementary Materials.

## Supplementary Materials

The following supporting information can be downloaded at: https://www.mdpi.com/article/10.3390/ani15152312/s1. Figure S1. The maximum likelihood phylogeny of PEDV strains. Phylogenetic reconstruction of 556 PEDV sequences, encompassing classical G1 strains, pandemic G2 reference strains, and S-INDEL variants. Branches are color-coded according to genotype. Genotype-discriminating residues (positions 55–65 and 166–172) are annotated adjacent to corresponding genotype. Figure S2. The recombination analysis of PEDV S-INDEL strains. Recombination events detected in S-INDEL strains using RDP5 (*p* < 0.01, ≥3 methods). Events are classified as inter- or intra-genotypic based on parental lineage origins. Figure S3. Differential residue frequencies across S1 protein. Red triangles represent each amino acid mutation site. X-axis: amino acid position and identity; Y-axis: occurrence frequency (%) per clade. Figure S4. The maximum clade credibility (MCC) phylogeny of PEDV S-INDEL strains. Branches colored according to continent of collection location. Table S1. Sequence information used in study. Table S2. Recombination results of S-INDEL strains with other genotypes detected with RDP v5.67. Table S3. Recombination results between clade 1 and clade 2 S-INDEL strains detected with RDP v5.67. Table S4. Positive sites detected with different methods.
