# Supplementary material for: Clade-Specific Recombination and Mutations Define the Emergence of Porcine Epidemic Diarrhea Virus S-INDEL Lineages
Source: Animals (Basel). 2025 Aug 7;15(15):2312. doi: 10.3390/ani15152312 (PMC12345761; doi:10.3390/ani15152312)
Supplement: Supplementary file 1 [file animals-15-02312-s001.zip › Table S4.pdf]

Table S4. Positive sites detected with different methods.

codon	FEL	SLAC	MEME	FUBAR
	P value	*P* value	*P* value	posterior probability
10	0.0221	>0.1	0.033	0.015
83	0.0000	0.000	0	0
113	0.0083	0.023	0	0
114	0.0128	0.052	0.017	0
156	0.0858	0.034	0	0.004
309	0.0127	0.026	0.02	0.002
609	0.0134	0.040	0.021	0.001
