# Supplementary figures and images for: Clade-Specific Recombination and Mutations Define the Emergence of Porcine Epidemic Diarrhea Virus S-INDEL Lineages
Source: Animals (Basel). 2025 Aug 7;15(15):2312. doi: 10.3390/ani15152312 (PMC12345761; doi:10.3390/ani15152312)

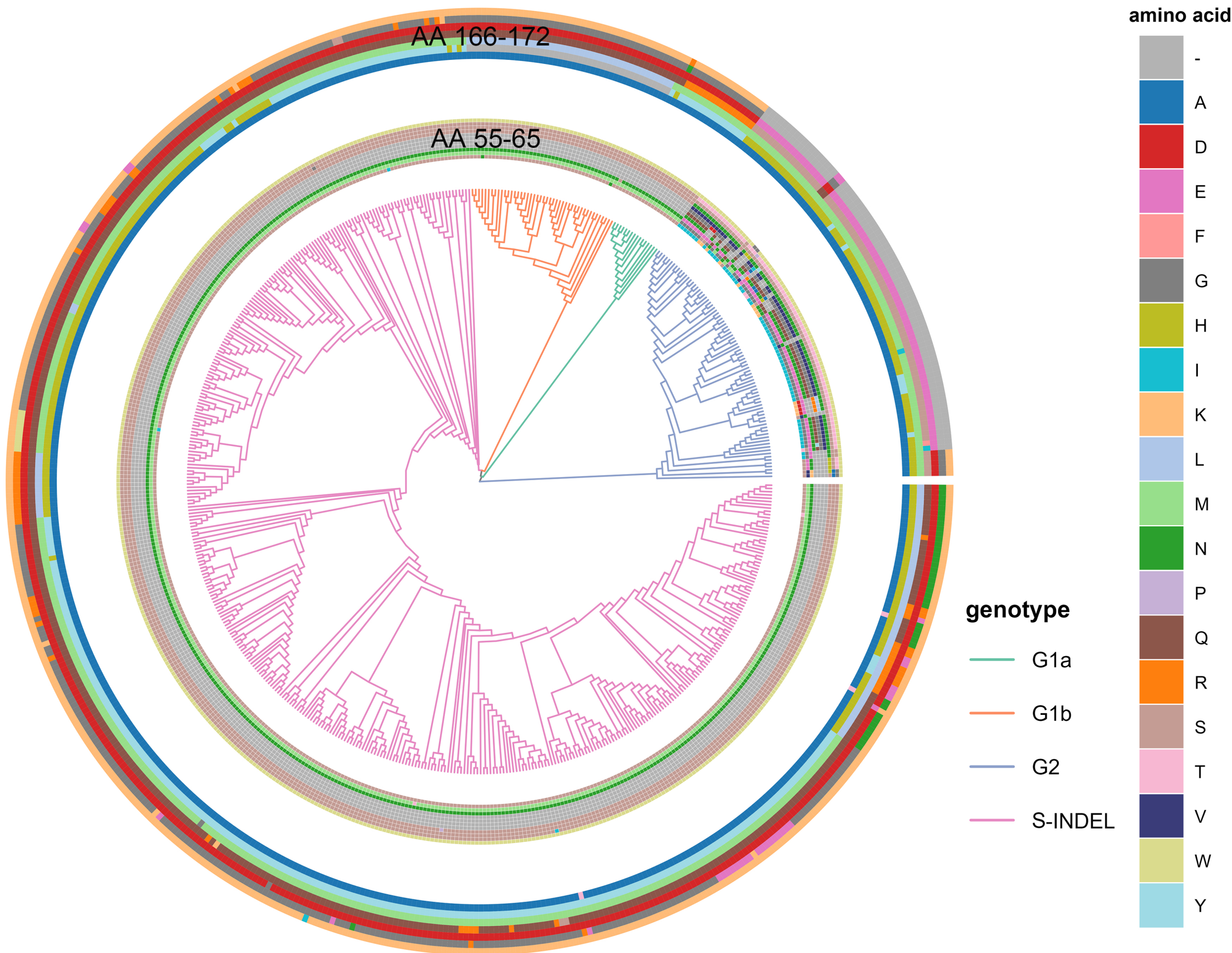

Supplement: Supplementary file 1 [file animals-15-02312-s001.zip › Figure S1.pdf]

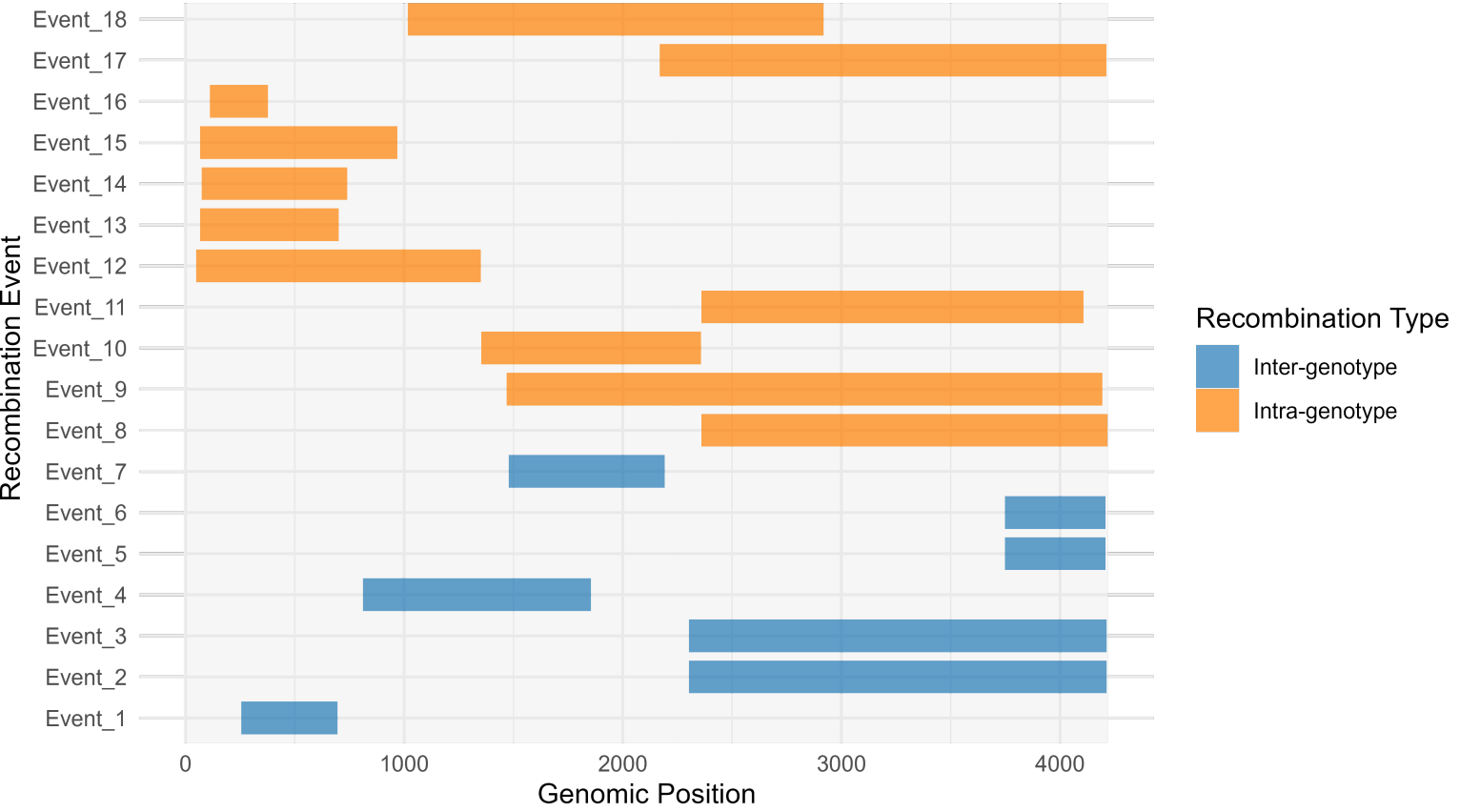

Supplement: Supplementary file 1 [file animals-15-02312-s001.zip › Figure S2.pdf]

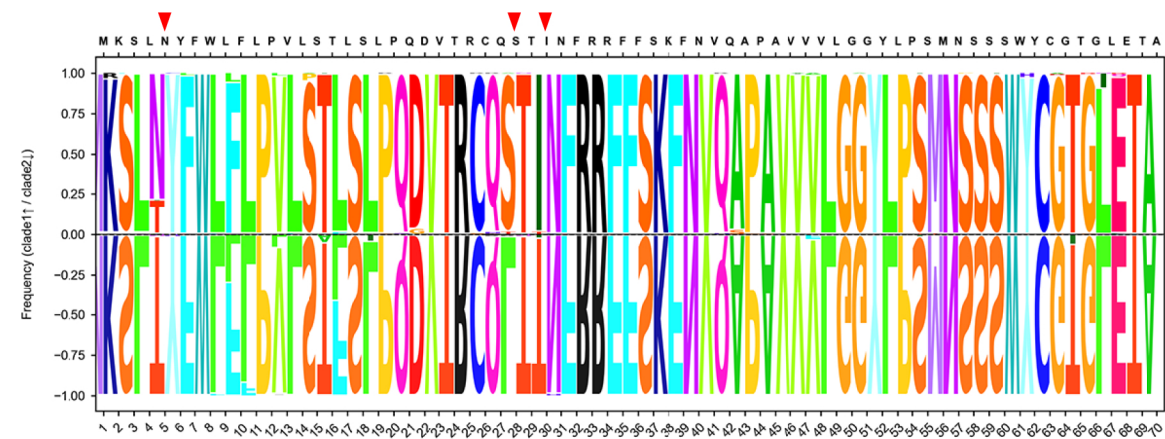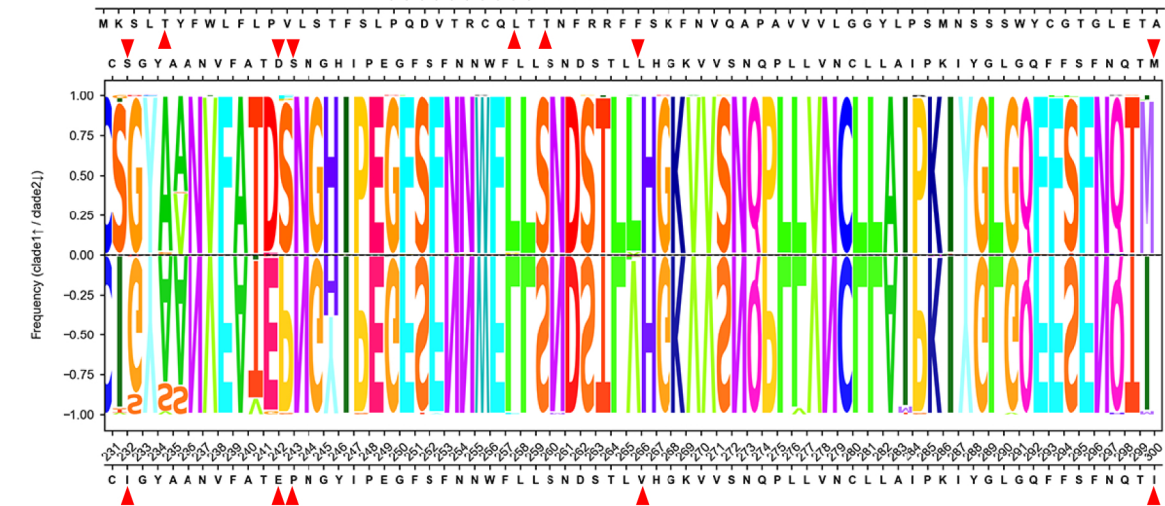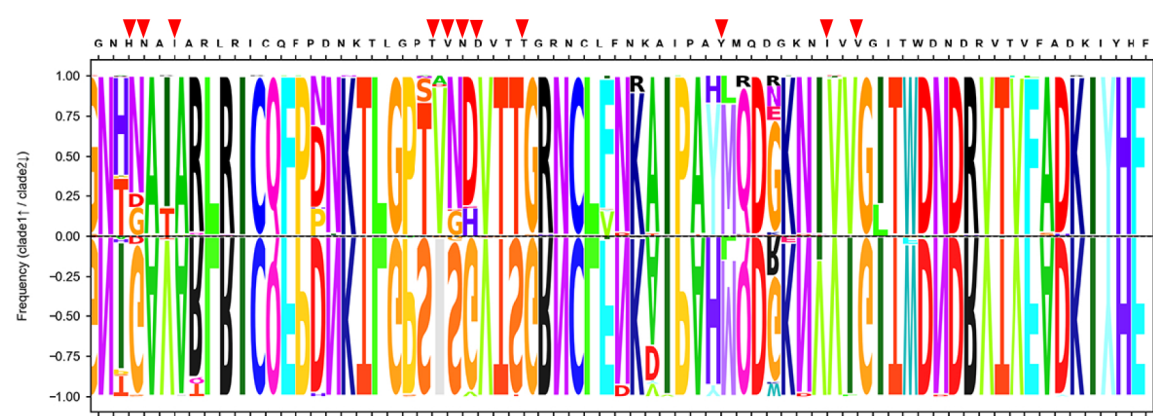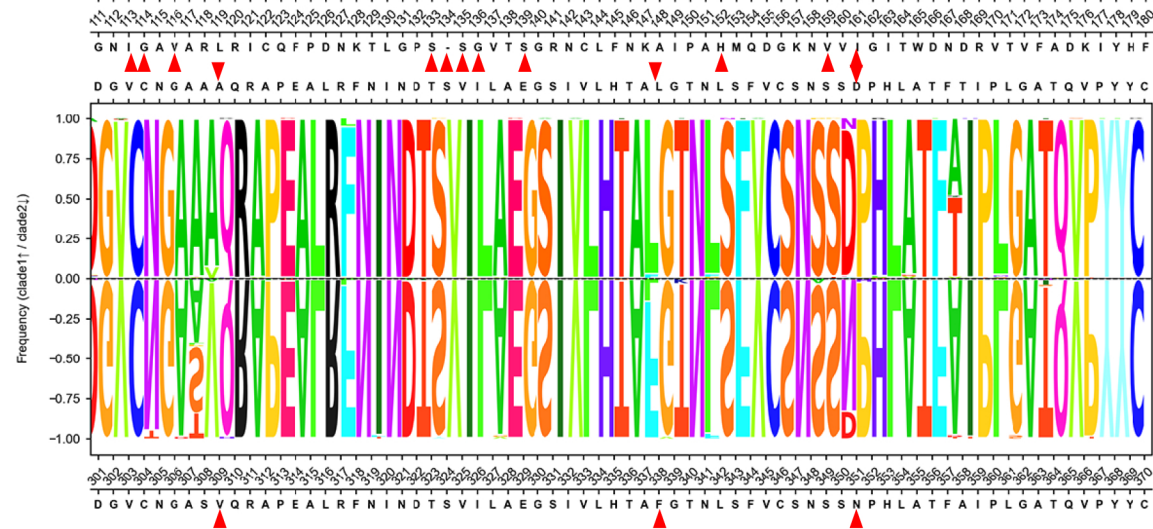

Supplement: Supplementary file 1 [file animals-15-02312-s001.zip › Figure S3.pdf]

location

- Oceania
- Europe
- South America
- North America
- Asia

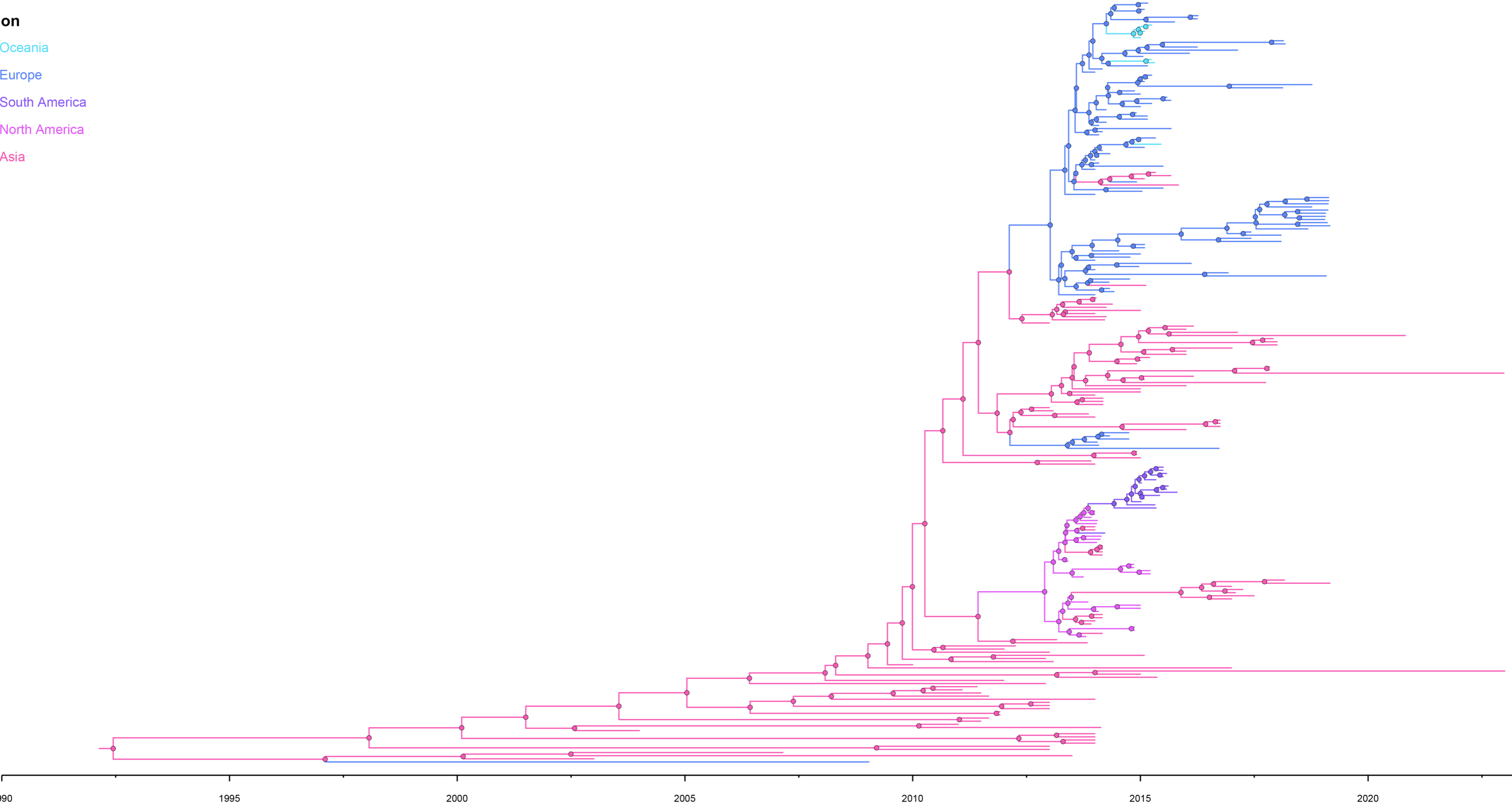

Supplement: Supplementary file 1 [file animals-15-02312-s001.zip › Figure S4.pdf]
